# The impact of the L-dopa test on cortisol response and its comparison with the insulin tolerance test (comparative clinical study)

**DOI:** 10.1097/MD.0000000000044442

**Published:** 2025-09-05

**Authors:** Fatma Özgüç Çömlek, Hümeyra Yaşar Köstek, Emine Dilek, Filiz Tütüncüler Kökenli

**Affiliations:** a Division of Pediatric Endocrinology, School of Medicine, Trakya University, Edirne, Türkiye; b Division of Pediatric Endocrinology, School of Medicine, Selçuk University, Konya, Türkiye.

**Keywords:** cortisol, HPA axis, insulin tolerance test, L-dopa test

## Abstract

Insulin tolerance tests (ITTs), glucagon tests, and low or standard-dose adrenocorticotropic hormone tests are used to evaluate the hypothalamic–pituitary–adrenal (HPA) axis. While ITT is considered the gold standard test for stimulating both the cortisol and growth hormone axis, its use in young children carries a risk of hypoglycemia, making it potentially unsafe. Recent studies indicate that L-dopa stimulates the release of adrenocorticotropic hormone and cortisol. This study aimed to evaluate the L-dopa test-evoked cortisol response in short children and compare it with the ITT. L-dopa and ITTs were used as growth hormone stimulation tests. Blood samples were collected from the subjects at 0, 30th, 60th, 90th, and 120th minutes to analyze growth hormone and cortisol levels. A serum cortisol level of >18 µg/dL in both tests was considered an adequate response for the HPA axis. All cases (100%) in the ITT showed a peak cortisol response more significant than 18 µg/dL. In comparison, an adequate cortisol response was achieved in 18 cases (90%) in the L-dopa test. When the time to reach peak cortisol levels was evaluated, in the L-dopa test, it was at the 120th minute in 70% of the cases (14 cases), and in the ITT, it was at the 60th minute in 65% of the cases (13 cases). The L-dopa stimulation test effectively controlled the HPA axis and stimulated cortisol levels similarly to ITT. In situations where the use of ITT is risky, the L-dopa test can be employed to monitor the HPA axis.

## 1. Introduction

Various physiological and pharmacological factors can stimulate cortisol secretion in humans. Nearly any type of physical stress, such as prolonged exercise or severe trauma, increases circulating cortisol levels due to heightened pituitary adrenocorticotropic hormone secretion.^[1]^ Insulin-induced hypoglycemia (IIH) is the most reliable method for evaluating the integrity of the hypothalamic–pituitary–adrenal (HPA) axis.^[2]^

In children, growth hormone deficiency (GHD) is identified through careful clinical evaluation and confirmed by conducting 1 or more growth hormone stimulation tests. There is considerable experience in assessing growth hormone (GH) production in pediatric populations using provocative tests with stimuli such as IIH, arginine, L-dopa, clonidine, and glucagon. While the IIH test was once deemed the gold standard, it poses risks and necessitates rigorous medical supervision in a controlled clinical environment. Consequently, it has been mostly supplanted by the glucagon test.^[3,4]^ However, the glucagon test has several disadvantages, including a lengthy duration of 180 minutes and potential side effects such as vomiting and hypoglycemia.^[5]^

The L-dopa test is a straightforward procedure used to investigate suspected GHD. Recent studies indicate that L-dopa, traditionally used for ~50 years to assess GHD, can also aid in diagnosing adrenal insufficiency by stimulating cortisol secretion.^[6]^ In this study, we aimed to evaluate the effectiveness of the L-dopa test as a stimulant for cortisol secretion in short children and to compare cortisol secretion after L-dopa stimulation with that following insulin tolerance test (ITT) stimulation.

## 2. Materials and methods

This single-center, cross-sectional study included patients who applied to the Trakya University Medical Faculty Pediatric Endocrinology Clinic due to short stature and whose height was <−2 SD for age and gender.

All patients underwent a thorough physical examination and laboratory evaluation, including thyroid function tests, to measure possible endocrine pathology. Patient data, including clinical, anthropometric, and laboratory information, were gathered from medical records. Patients with chronic diseases, such as gastrointestinal, cardiovascular, and respiratory issues, those with known endocrine disorders (particularly Cushing syndrome and hypothyroidism), individuals with a history of medication use (including steroids and antipsychotics), or those suspected of having a deficiency in more than 1 pituitary hormone were excluded from the study. A Harpenden height meter with a precision of 0.1 cm was used to measure height. The National Growth Chart for Turkish children was used for an auxological evaluation.^[7]^ This study was conducted following the Declaration of Helsinki Ethical Principles. It was approved by the Trakya University Medical Faculty Clinical Research Ethics Committee. All participants in the study were informed about its substance, and written consent was obtained from their parents. All experiments were conducted as routine diagnostic procedures between 7:30 and 8:00 am, following a 10- to 12-hour fasting period. L-dopa and ITTs were used as GH stimulation tests. For the L-dopa test, a single dose of L-dopa (Madopar) was administered orally according to body weight (BW > 30 kg; 500 mg, 15–30 kg; 250 mg, <15 kg; 125 mg). Blood samples were collected from the subjects at 0, 30th, 60th, 90th, and 120th minutes to analyze GH and cortisol levels. ITT was conducted as a second stimulation test in cases where serum GH levels were <10 ng/mL. For the ITT, crystallized insulin at 0.1 U/kg dose was administered intravenously. In cases where blood glucose levels are below 40 mg/dL, this condition is considered hypoglycemia. The ITT application collected blood samples at 0, 30th, 60th, 90th, and 120th minutes, and blood sugar, GH, and cortisol levels were measured.

All samples were analyzed right away. A serum cortisol level of >18 µg/dL in both tests was considered an adequate response for the HPA axis.

### 2.1. Statistical analyses

The SPSS software package for Windows (Version 20.0; SPSS Inc., Chicago) was used for statistical data analysis. The Kolmogorov–Smirnov test was used to evaluate the distribution of the data. In numerical comparisons, the Student *t* test or Mann–Whitney *U* test was used to evaluate the differences between 2 groups according to the conformity of the measured parameters to normal distribution. Spearman rho correlation was employed to assess the relationships between the variables. Wilcoxon Signed Rank test was used to compare prepubertal and pubertal groups. While categorical data were expressed as frequency (%), numerical data were expressed as mean ± standard deviation. In all statistical tests, *P*-values <.05 were considered significant.

## 3. Results

Twenty children (12 boys and 8 girls) were included in the study.The mean age of the cases was 11.7 ± 2.9 years, and 10 cases (50%) were in the pubertal period. The mean height Standard Deviation Score (SDS) of the cases was −2.97 ± 0.78, the mean body weight SDS was −1.62 ± 1.03, and the mean body mass index (BMI) SDS was −0.22 ± 1.03.

Although the mean peak cortisol level in the L-dopa stimulation test was 24.2 ± 6.2 µg/dL and the mean peak cortisol response in the ITT was 26.2 ± 4.0 µg/dL, the difference was not significant (*P* = .26). The average cortisol response in both tests was >18 µg/dL. In the L-dopa test, the mean peak GH level was 4.3 ± 2.1 ng/mL, while in the ITT, it was 5.7 ± 2.7 ng/mL, and the difference was not significant (*P* = .06) (Table 1). All cases (100%) in the ITT showed a peak cortisol response more significant than 18 µg/dL, while an adequate cortisol response was achieved in 18 cases (90%) in the L-dopa test. The peak cortisol levels of the 2 cases with peak cortisol levels <18 µg/dL with the L-dopa test were 13.7 µg/dL and 14.4 µg/dL, respectively, while the peak cortisol levels of these cases with the ITT test were 21.9 µg/dL and 31.3 µg/dL, respectively. When the time to reach peak cortisol levels was evaluated, in the L-dopa test, it was at the 120th minute in 70% of the cases (14 cases), and in the ITT, it was at the 60th minute in 65% of the cases (13 cases). GH deficiency was diagnosed in 95% (19 cases) of the cases that underwent GH stimulation test. In pubertal cases, mean peak growth hormone response with ITT (6.8 ± 2.6 ng/mL) was significantly higher than the response with L-dopa test (4.3 ± 2.7 ng/mL) (*P* = .02, Table 2).

**Table 1:** Comparison of peak growth hormone and cortisol levels in stimulation tests and pubertal status of the cases.

n (%)	Peak growth hormone response (ng/mL) (mean ± SD)			Peak cortisol response (µg/dL) (mean ± SD)		
	L-dopa	ITT	*P*	L-dopa	ITT	*P* *
Prepubertal, n = 10 (50%)	4.3 ± 1.5	4.6 ± 2.4	.79	25.4 ± 5.1	25.5 ± 4.7	.87
Pubertal, n = 10 (50%)	4.3 ± 2.7	6.8 ± 2.6	.02	23.1 ± 7.3	26.8 ± 3.4	.13

GH = growth hormone, SD = standart deviation.

* The Wilcoxon signed-rank test was used to compare prepubertal and pubertal groups.

**Table 2:** Times to reach peak cortisol levels with L-dopa stimulation test and ITT.

Time (min)	L-dopa, n (%)	ITT, n (%)
0	1 (5)	0 (0)
30	4 (20)	1 (5)
60	0 (0)	13 (65)
90	1 (5)	3 (15)
120	14 (70)	3 (15)

ITT = insulin tolerance test.

## 4. Discussion

Study results showed that L-dopa is a potent stimulator of cortisol secretion in short children and can be used to assess HPA axis integrity. By comparing the cortisol responses to L-dopa and ITTs, we demonstrated that, despite some variabilities among patients, L-dopa is comparable in effectiveness to ITT. The finding supports that 90% of children had a normal cortisol response following L-dopa stimulation.

Dopamine is the primary catecholamine neurotransmitter in the central nervous system. It regulates various functions, including cognition, emotion, hunger, satiety, and the endocrine system, such as prolactin suppression. Although dopamine cannot cross the blood-brain barrier directly, L-dopa, its precursor, can and binds to dopamine receptors (D1–D5) in the central nervous system, facilitating its functions.^[8]^ The administration of the dopamine agonist bromocriptine has been shown to increase proopiomelanocortin mRNA levels in the pituitary gland and hypothalamus, which is the prohormone of adrenocorticotropic hormone.^[9]^ Another study has proposed that L-dopa is converted to norepinephrine by dopamine β-hydroxylase, which positively regulates the HPA axis by stimulating corticotropinreleasing hormone.^[10]^ Similar to previous studies, we obtained a sufficient response with L-dopa stimulation, identical to ITT in our research.^[6,11]^

The maturation of the HPA axis is completed with age and the progression of puberty, leading to an increase in cortisol secretion.^[12,13]^ It has been reported that age and pubertal status, as well as BMI, may affect the GH and cortisol response to tests.^[14–16]^ This study found no correlation between peak cortisol level and pubertal status or age. On the contrary, 2 patients who underwent L-dopa testing and had cortisol levels <18 µg/dL were in the pubertal period. In our study, there were no obese children with BMI > 2 SD.

During L-dopa testing, the peak cortisol response was generally achieved at 120 minutes.^[6,11]^ This current study did not consider the factors that influence the bioavailability of oral L-dopa and, consequently, the peak cortisol levels. However, peak cortisol levels were generally observed at the 120th minute of the test, consistent with previous studies. Considering these findings, evaluating the HPA axis with L-dopa by taking blood only at the 120th minute may be suggested under resource-constrained conditions.

Our findings also indicated that the L-dopa test produced a satisfactory cortisol response, comparable to the gold standard ITT. It has been reported that ITT is unsafe and occasionally causes deaths and neurological complications in children.^[4,17]^

In previous studies, some reported inadequate cortisol response to the L-dopa test. In contrast, others stated that administering propronal together with L-dopa would increase the stimulating effect of L-dopa.^[18,19]^ However, in this study, the L-dopa test obtained successful results similar to those of the ITT tests, which are considered the gold standard in evaluating the HPA axis.

L-dopa is known to produce a high rate of false-negative results and is typically used alongside arginine to enhance sensitivity in growth hormone stimulation tests.^[20]^ In our clinical practice, we evaluate our patients whose peak GH levels are insufficient with L-dopa with a second GH stimulation test, which is usually ITT. Indeed, in this study, GH deficiency was excluded in the ITT test in 1 patient with insufficient GH response to L-dopa. In this way, we compared the success of ITT and L-dopa tests in HPA axis evaluation.

The number of patients participating in the study and the side effects experienced by the patients during the test application, especially the fact that physiological stresses such as nausea and vomiting, which are commonly seen in the L-dopa test, may have stimulated adrenocorticotropic hormone, were not taken into account in the design of the study, and can be considered as limitations of the study. However, we believe that this study makes an essential contribution to the literature, as similar studies have been conducted with limited patient numbers and comparisons with different tests.

In conclusion, the L-dopa stimulation test effectively controls the HPA axis and stimulates cortisol levels similarly to ITT. The peak cortisol response time in the L-dopa test was initially determined to be 120 minutes. In cases where ITT is risky, the L-dopa test can be used to control the HPA axis. On the other hand, in cases of puberty, ITT may be more effective than the L-dopa test in controlling the GH axis.

## Author contributions

**Conceptualization:** Fatma Özgüç Çömlek, Filiz Tütüncüler Kökenli.

**Data curation:** Fatma Özgüç Çömlek, Hümeyra Yaşar Köstek, Filiz Tütüncüler Kökenli.

**Formal analysis:** Fatma Özgüç Çömlek.

**Funding acquisition:** Fatma Özgüç Çömlek, Hümeyra Yaşar Köstek.

**Investigation:** Fatma Özgüç Çömlek.

**Methodology:** Fatma Özgüç Çömlek.

**Project administration:** Fatma Özgüç Çömlek, Emine Dilek.

**Resources:** Fatma Özgüç Çömlek, Hümeyra Yaşar Köstek.

**Software:** Fatma Özgüç Çömlek.

**Supervision:** Fatma Özgüç Çömlek, Emine Dilek.

**Validation:** Hümeyra Yaşar Köstek, Filiz Tütüncüler Kökenli.

**Visualization:** Fatma Özgüç Çömlek, Emine Dilek.

**Writing – original draft:** Fatma Özgüç Çömlek, Hümeyra Yaşar Köstek, Filiz Tütüncüler Kökenli.

**Writing – review & editing:** Fatma Özgüç Çömlek, Filiz Tütüncüler Kökenli.
